# A realist review of how, why, for whom and in which contexts quality improvement in healthcare impacts inequalities

**DOI:** 10.1136/bmjqs-2024-017386

**Published:** 2025-01-19

**Authors:** Lucy Lara Johnson, Geoff Wong, Isla Kuhn, Graham P Martin, Anuj Kapilashrami, Laura Lennox, Georgia Bell Black, Matthew Hill, Ryan Swiers, Hashum Mahmood, Linda Jones, Jude Beng, John Ford

**Affiliations:** 1 Queen Mary University of London Wolfson Institute of Population Health, London, UK; 2 Primary Care Health Sciences, University of Oxford, Oxford, UK; 3 THIS Institue, University of Cambridge, Cambridge, UK; 4 THIS Institute, University of Cambridge, Cambridge, UK; 5 School of Health and Social Care, University of Essex, Colchester, UK; 6 CLAHRC for Northwest London, London, UK; 7 School of Public Health, Imperial College London, London, UK; 8 Q Community, The Health Foundation, London, UK; 9 South Tyneside and Sunderland NHS Foundation Trust, South Shields, UK; 10 NHS Confederation, London, UK; 11 Patient and Public Involvement Representative, Sheffield, UK

**Keywords:** Quality improvement, Health policy, Healthcare quality improvement

## Abstract

**Introduction:**

Quality improvement (QI) is aimed at improving care. Equity is one of the six domains of healthcare quality, as defined by the Institute of Medicine. If this domain is ignored, QI projects have the potential to maintain or even worsen inequalities.

**Aims and objectives:**

We aimed to understand why, how, for whom and in which contexts QI approaches increase, or do not change health inequalities in healthcare organisations.

**Methods:**

We conducted a realist review by first developing an initial programme theory, then searching MEDLINE, Embase, CINAHL, PsychINFO, Web of Science and Scopus for QI projects that considered health inequalities. Included studies were analysed to generate context-mechanism-outcome configurations (CMOCs) and develop an overall programme theory.

**Results:**

We screened 6259 records. Thirty-six records met our inclusion criteria, the majority of which were from the USA. We developed CMOCs covering four clusters: values and understanding, resources, data, and design. Five of these described circumstances in which QI may increase inequalities and 15 where it may reduce inequalities. We found that QI projects that are values-led and incorporate diverse, patient-led data into design are more likely to address health inequalities. However, when staff and patients cannot engage fully with equity-focused projects, due to practical or technological barriers, QI projects are more likely to worsen inequalities.

**Conclusions:**

The potential for QI projects to positively impact inequalities depends on embedding equity-focused values across organisations, ensuring sufficient and appropriate resources are provided to staff delivering QI, and using diverse disaggregated data alongside considered user involvement to inform and assess the success of QI projects. Policymakers and practitioners should ensure that QI projects are used to address inequalities.

**WHAT IS ALREADY KNOWN ON THIS TOPIC**

- Quality improvement (QI) projects can worsen, maintain or improve health inequalities.
- However, we do not know why, how, for whom and in which contexts QI approaches increase, decrease or do not change health inequalities in healthcare organisations.

**WHAT THIS STUDY ADDS**

- This study is the first realist review to explore the impact of QI on health and care inequalities.
- We developed context-mechanism-outcome configurations (CMOCs) that describe and explain the conditions under which QI projects can contribute to health inequalities or improve them.
- We organised these CMOCs into four clusters: values and understanding, resources, data, and design.

**HOW THIS STUDY MIGHT AFFECT RESEARCH, PRACTICE OR POLICY**

- Through our programme theory and CMOCs, we provide examples of when, why and for whom equity-focused QI occurs.
- These results can help to guide policymakers and practitioners to embed a health equity focus in QI projects in healthcare organisations.

## Introduction

 Healthcare quality is not a static concept, but one that varies according to context.^1^ Over 20 years ago, the Institute of Medicine provided a broad definition of quality still used today. Quality care is safe, effective, patient-centred, timely, efficient and equitable.^2^ Since then, a growing international body of literature has emphasised the importance of assessing inequalities in healthcare provision and undertaking quality improvement (QI) that promotes health equity.^3^,^10^ We follow the Robert Wood Johnson Foundation definition of health equity—‘everyone has a fair and just opportunity to be as healthy as possible’.^11^ In the context of QI, this means that everyone has the opportunity to receive the same high-quality healthcare irrespective of their background, which usually means intervening to ensure that resources are allocated proportionate to need.

If equity is not central to QI, there can be unintended consequences.^12^ Health and care inequalities, the unfair and avoidable differences in health and care across the population and between different groups within society,^13^ can arise if all patients do not benefit equally from projects. For example, in the 1990s New York State began publishing coronary artery bypass graft mortality report cards to enable patients to choose the highest performing surgeons and hospitals, while incentivising healthcare providers to improve care and consequently their mortality data.^14^ Areas with the report card saw increased racial and ethnic inequalities, compared with areas without the report card, with white patients receiving more operations than their black or Hispanic counterparts. It took 9 years for inequalities in surgery to return to preimplementation levels.

In the current literature, some authors have advocated for specific equity-focused QI (EFQI) approaches,^3^ whereas others argue that all QI interventions must take a health equity perspective to reduce health inequalities.^6^ To make QI more equitable, we need a better understanding of how QI may impact health and care inequalities. We aimed to explore why, how, for whom and in which contexts QI approaches increase, decrease or do not change inequalities in healthcare organisations.

## Methods

We undertook a realist review of published QI studies that reported impacts on health and care inequalities. A realist review is a theory-driven evidence synthesis that focuses on explaining how and why things happen.^15^ It can provide policymakers and practitioners with practical understandings of ‘what works’ for whom, when, how and why within complex social interventions^16^ by identifying causal explanations within research papers and other documentary sources of evidence. These causal explanations are articulated in the form of context-mechanism-outcome configurations (CMOCs). It is ideally suited to this research question because it provides the methodology to understand how and why QI interventions address (or do not address) health and care inequalities, rather than seeking to arrive at a binary conclusion about whether QI projects increase or decrease inequalities.

**Figure 1:**
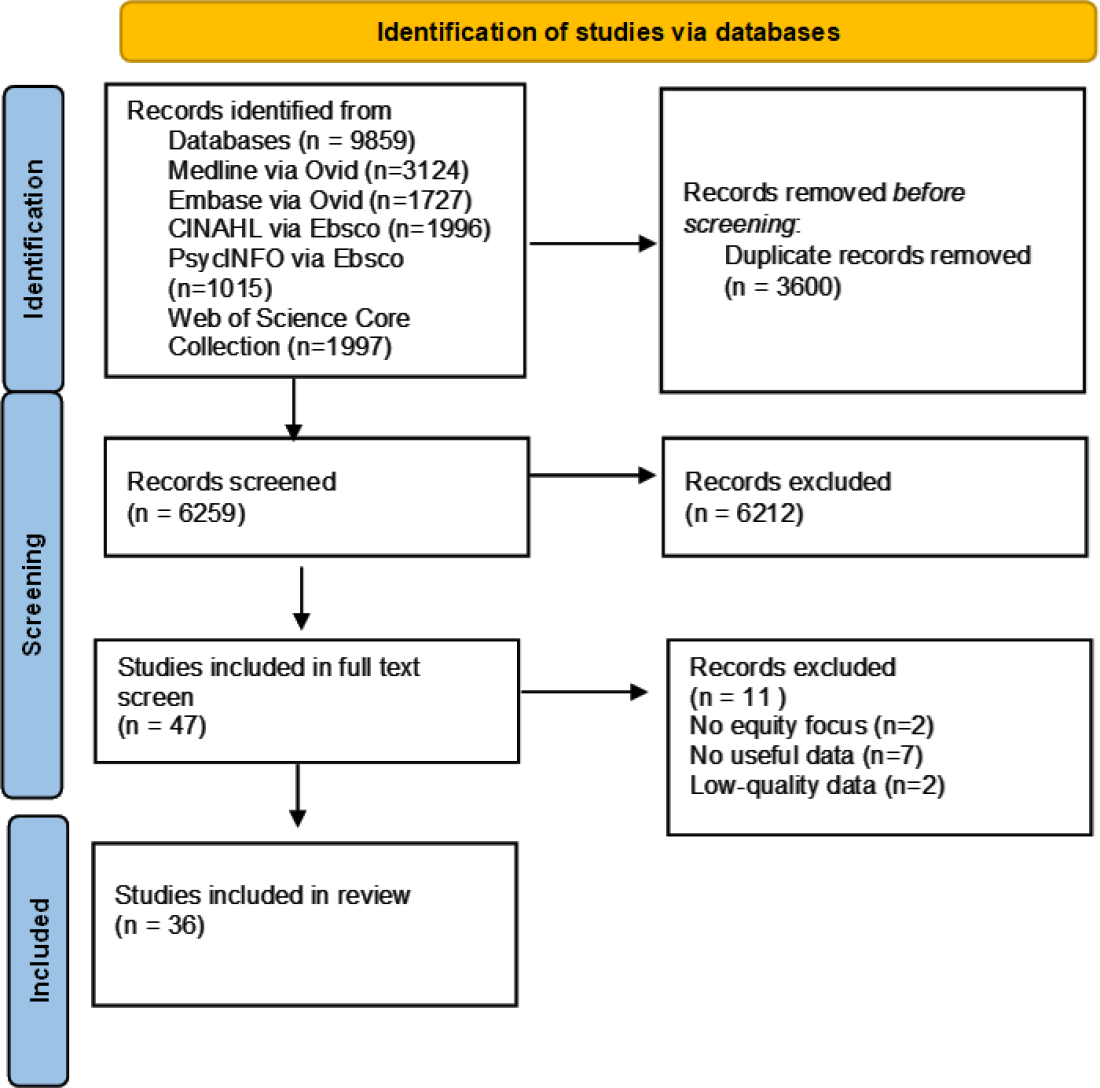
Preferred Reporting Items for Systematic Reviews and Meta-Analyses (PRISMA) diagram.

The review was registered with PROSPERO (CRD42023381062). We developed an initial programme theory (IPT) drawing on literature identified during grant funding development and through a workshop with our expert panel. The IPT guided the search strategy by ensuring that key concepts were included. The IPT was iteratively refined during the review into the final programme theory (figure 1). We searched MEDLINE (Medical Literature Analysis and Retrieval System Online), Embase, CINAHL (Cumulative Index to Nursing and Allied Health Literature), PsychINFO, Web of Science and Scopus to identify QI studies that reported health or care inequalities published between January 2000 and January 2023 (see online supplemental appendix 1 for full search terms).

The title and abstracts of each record were screened by LLJ using Rayyan,^17^ according to the eligibility criteria outlined in table 1. We included any QI article that included data relating to impact on disadvantaged groups. The authors of the article had to identify the intervention as a QI initiative to meet the eligibility criteria.

**Table 1:** Inclusion and exclusion criteria

Inclusion criteria	Exclusion criteria
Articles in healthcare settings describing or evaluating QI projects or initiatives AND Articles that report on the impact of the QI initiative on closing the health gap according to the PROGRESS Plus groups^22^ (see Table 2) OR focusing on specific disadvantaged groups (eg, low-income or minority ethnic groups) or inclusion health groups (eg, people who are homeless) AND Any study design AND Any clinical topic in any healthcare setting	Studies that focus on specific disadvantaged groups (eg, low-income or minority ethnic groups) or inclusion health groups (eg, people who are homeless) but do not look at the inequality gap OR Studies undertaken in low-income or middle-income countries, as defined by the OECD (the Organisation for Economic Co-Operation and Development) OR Not published in English OR Protocols, editorials, conference proceedings, and opinion pieces OR Published before 2000

QI, quality improvement.

During the screening process, we tagged screened articles according to PROGRESS Plus criteria to ensure that included papers had health equity data. PROGRESS Plus is an acronym used to guide equity-focused analysis during systematic reviews^18^ (table 2). During the screening process, we further categorised papers by whether the intervention was aimed at closing the gap, that is, focused on narrowing inequalities between PROGRESS Plus groups (tagged ‘gap’), or targeted specific disadvantaged groups by focusing on their specific needs (tagged ‘targeted’). A random sample of 10% of these papers was screened by JF to check for systematic inconsistencies. Any disagreements were resolved through discussion between LLJ and JF.

**Table 2:** PROGRESS Plus criteria

	Refers to:
P	Place of residence
R	Race/ethnicity/culture/language
O	Occupation
G	Gender/sex
R	Religion
E	Education
S	Socioeconomic status
S	Social capital
Plus	Personal characteristics associated with discrimination (eg, age, disability) Features of relationships (eg, smoking parents, excluded from school) Time-dependent relationships (eg, leaving the hospital, respite care, other instances where a person may be temporarily at a disadvantage)

During the full-text screening process, documents were assessed for relevance and rigour. This involved assessing documents and sections of text according to the relevance and robustness of the methodological approach used to generate the data.^19^

Document characteristics were detailed in Excel. Included documents were uploaded to NVivo V.12 for coding. Codes were arrived at deductively (drawn from the IPT) and inductively (identified during the analysis process) and organised into ‘parent nodes’ (eg, ‘barriers to implementation’) with subsequent ‘child nodes’ (eg, ‘staff communication’, ‘patient navigation’). Each included study was coded and then recoded.

We synthesised data within codes into CMOCs through interpretative cross-case comparison. Realist analysis is exploratory, and aims to refine theories about what works, for whom, why and in what circumstances.^16^ We explored data present across the full text of the documents, to compare interventions and understand how and why certain outcomes occurred in certain contexts (see online supplemental appendix 2). This cross-case comparison was important, as often not all parts of a CMOC were articulated in the same document. Instead, we consolidated and juxtaposed our data to develop CMOCs that were evidenced in multiple documents. We used a series of questions relating to the relevance and rigour of content within data sources as part of our process of analysis and synthesis:

- Relevance: Are sections of text within this document relevant to programme theory development?
- Rigour (judgements about trustworthiness): Are these data sufficiently trustworthy to warrant making changes to any aspect of the programme theory?
- Interpretation of meaning: If the section of text is relevant and trustworthy enough, do its contents provide data that may be interpreted as functioning as context, mechanism or outcome?
- Interpretations and judgements about CMOCs: How are contexts, mechanisms or outcomes configured into a CMOC? Are there further data to inform the CMOCs contained within this document, or other documents? If so, which other documents? How does this CMOC relate to other CMOCs that have already been developed?
- Interpretations and judgements about programme theory: How does the (full or partial) CMOC relate to the programme theory? Within this same document, are there data which inform how the CMOC relates to the programme theory? If not, are there data in other documents? Which ones? Considering this CMOC and any supporting data, does the programme theory need to be changed?

We developed and refined the CMOCs into an overall programme theory. In addition, several substantive theories informed our analytical process. For example, Cluster Four (‘Design’, see table 3) was influenced by the service user and public involvement literature.^20^,^22^ We identified these theories through previous knowledge of the literature, alongside an extended informal literature search that purposively sought out potentially relevant theories. The programme theory was developed through this process alongside engagement with substantive theories and through iterative conversations with our expert panel, academics, clinicians, and our project’s patient and public involvement (PPI) group. Specifically, the PPI group met every month and supported the design of the IPT and interpretation of emerging findings.

**Table 3:** Context-mechanism-outcome configurations (CMOC)

	Cluster One: Values and Understanding
CMOC Number	CMOC
1A	When QI project developers are ‘vocal’ about the need for equity, and have knowledge about how inequity impacts health outcomes (C) then an ethical imperative for equity is embedded within QI projects (O) because they believe that equity constitutes a core part of QI (M)
1B	When QI developers have an emotive and collective determination to address existing and historic racial injustice in healthcare (C) projects that are designed and delivered to address these injustices (O) because there is an ethical imperative to do so (M)
2	When staff concerns question the ethics around targeting QI interventions at specific disadvantaged groups rather than all groups who need healthcare (C) it may be more difficult to do EFQI (O) because they have concerns about pursuing the problem with an *equity* focus rather than an *equality* focus (M)
3	When staff developing QI have a collective understanding of the wider determinants of health (C) they are more likely to design QI projects with fewer barriers that prevent disadvantaged patients from accessing them (O) because they have a commitment to social medicine
4	When QI staff work in an organisation that makes efforts to address implicit bias (C) then they are more likely to create EFQI projects (O) because they are more aware of their own implicit biases, and how these biases might contribute to the ‘root causes’ of health disparity(M)
	Cluster Two: Resources
5	When staff are given the time, training and resources to address inequalities in projects (C) they are more likely to consider the question of health equity (O) because they feel enabled to do so (M)
6	When staff tasked with time-consuming QI activity are also experiencing high workloads and administrative responsibilities (C) QI occurs via the most expeditious means (O) because staff have reduced capacity and/or bandwidth to engage with projects (M)
7A	When minority ethnic patients are provided with high-quality, multilingual, culturally competent resources and services (C) they are more likely to feel confident about the service (O) because they feel respected and supported (M)
7B	When staff are provided with adequate training to serve minority ethic patients (C) they are more likely to provide high-quality, equitable care (O) because they feel confident (M)
8	When staff have a lack of training, experience and exposure to the specific needs of diverse groups with different health needs (C) QI is designed and delivered for a preconceived idea of what the ‘average’ patient needs from a service (O) because staff lack awareness of the need to design and deliver EFQI (M)
	Cluster Three: Data
9	When organisations running QI have a culture of listening to, collating, analysing, and responding and reacting to the experiences of diverse staff and patients about the project (C) they are more likely to have a better understanding of the project’s impact (O) because of the wide range of knowledge gained (M)
10A	When the developers of QI projects have access to disaggregated granular data that enables them to identify the needs of specific people and communities (C) then they are more able to ensure that EFQI is being undertaken (O) because they have a better understanding of need in the population served (M)
10B	When the developers of QI projects have access to disaggregated granular data that enables them to monitor the progress of an EFQI project (C) then they are more able to ensure that EFQI is being undertaken (O) because they have a better understanding of the project’s impacts (M)
10C	When the developers of QI projects have access to disaggregated granular data that enables them to identify impacts on specific people (C) then they are more able to assess the health equity impact of the project itself (O) because they have a more holistic understanding of impact (M)
11A	When patient electronic records are incomplete (C) then EFQI is more difficult to carry out (O) because staff members struggle to find the correct patient data and to contact the patients most in need (M)
11B	When patient electronic records are difficult to combine and analyse (C) then EFQI is more difficult to carry out (O) because staff members have difficulty finding which individuals and patient groups more broadly are in need of specific services (M)
	Cluster Four: Design
12	When QI projects have and incorporate considerate representation of communities in which service user perspectives are given equal credence (C) QI projects will have a shared creation of knowledge, goals and services that are grounded in the lived experience of the target community (O) because they take on board the perspective of the community (M)
13	When QI projects involve multidisciplinary staff as equal partners in the design process (C) then QI initiatives will take a broad and inclusive approach (O) because a rich understanding of the multidimensional needs of patients is included in design (M)
14	When there is an ethos of co-creation embedded within the hospital and target patient groups and representations are deeply involved (C) then QI projects are more likely to have an equity focus (O) because mutual respect means that the voices of the target group are more likely to be heard (M)
15	When QI initiatives are designed such that they require a lot of patient agency, effort and engagement in order to gain the benefits (C) then those with the highest need and most significant challenges are the least likely to benefit from QI (O) because they most often lack the resources needed to engage with the initiative (M)

EFQI, equity-focused quality improvement; QI, quality improvement.

The review has been reported using RAMESES (Realist And Meta-narrative Evidence Syntheses: Evolving Standards) publication standards.^19^

## Results

### Overview of included documents

9859 records were identified using our search strategy. After the removal of duplicates, we screened 6259 records and excluded 6212. We performed a full-text screen of 47 records and excluded 11, leaving 36 articles included (figure 1).

**Figure 2:**
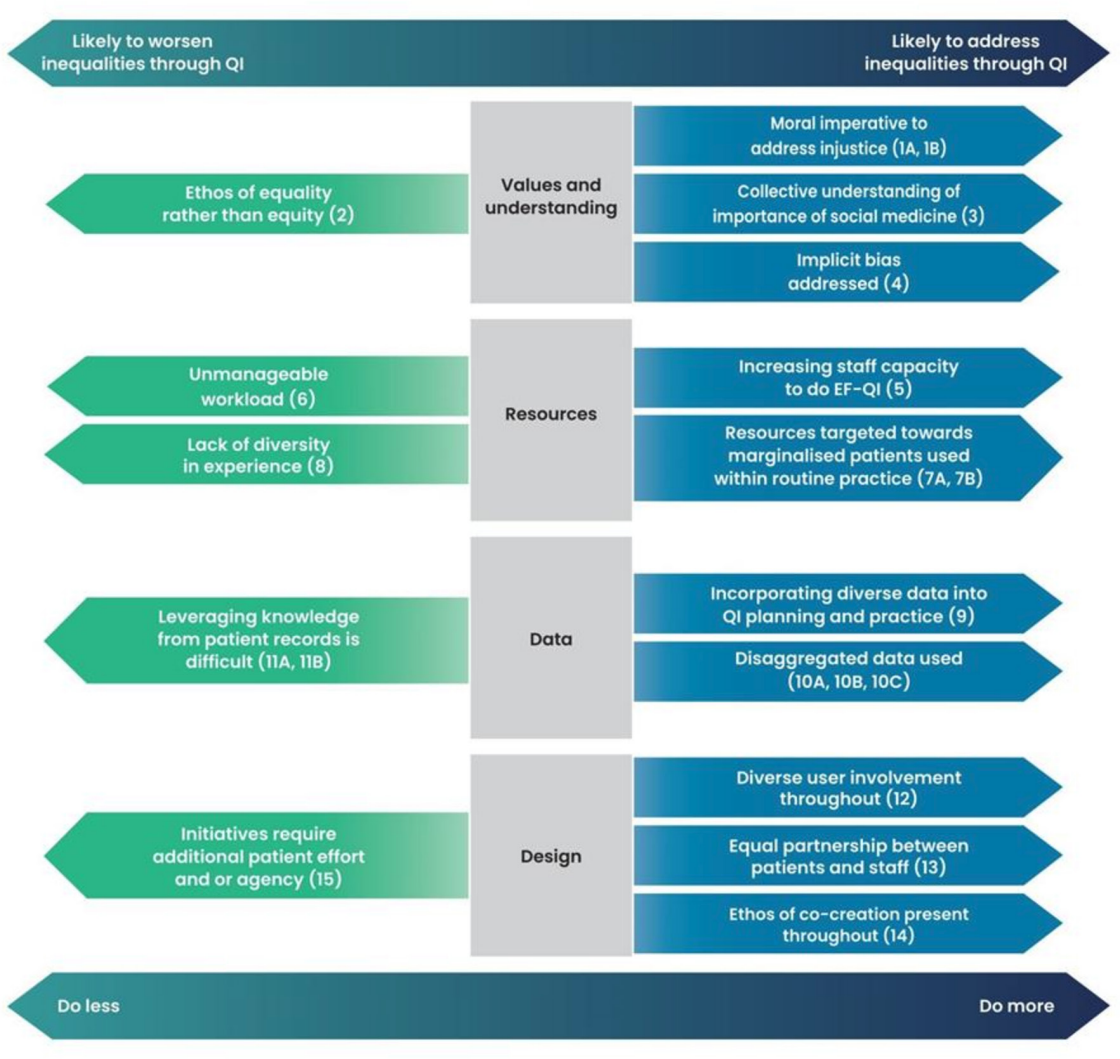
Programme theory diagram. QI, quality improvement; EFQI, equity-focused quality improvement.

Of these documents, 29 came from the USA, 5 from the UK, 1 from Ireland and 1 from Sweden (see online supplemental tables 1 and 2) (see online supplemental tables 1 and 2). Twenty-two documents investigated ‘gap’ interventions (online supplemental table 1) (online supplemental table 4), while 14 ‘targeted’ interventions (online supplemental table 2) (online supplemental table 5). Twelve documents covered primary care, 20 secondary care, 1 tertiary care and 3 community care. Most included documents (n=34) were published after 2010 and focused on more than one disadvantaged group (eg, race and gender). The most common PROGRESS Plus group was race/ethnicity/culture/language (n=23) followed by socioeconomic status (n=6) and gender (n=6). The ‘Plus’ category was included in 11 records, ranging from homeless populations to individuals living with severe mental illness.

### Context-mechanism-outcome configuration clusters

We developed CMOCs organised into four main CMOC clusters: values and understanding; resources; data; and design (table 3). These four CMOC clusters make up the four main components of our final programme theory (figure 2). There were 15 subthemes across each cluster. In total 20 CMOCs contributed to the overall programme theory; 15 CMOCs relate to reducing inequalities, and 5 CMOCs may worsen inequalities. Empirically, the included documents found a range of impacts on inequalities (see online supplemental table 4 and table 2). A list of CMOCs with examples from the documents can be found in online supplemental appendix 2.

#### Values and understanding

CMOCs 1A and 1B describe the moral imperatives that shaped QI work. Perhaps due to the large proportion of US articles in our data set, these moral imperatives often revolved around the concept of racial justice in healthcare. Many papers took the position that QI projects can be used to help tackle existing historical racial inequalities in health outcomes.^23^,^33^ Here there is an explicit value judgement: these inequalities are wrong, and healthcare professionals have a responsibility to improve them. We found that the moral or ethical values held by healthcare staff that relate to justice, equity and equality of healthcare provision can influence organisational/leadership culture and QI practice. Furthermore, a differential understanding of how healthcare quality is defined, and of the importance of the social determinants of health, leads to differences in how equity is considered in QI projects.

Within the data there were tensions between equality of healthcare provision and equity in QI projects (CMOC 2). For example, Burkitt *et al* describe the challenges faced during a QI project aimed at improving hypertension outcomes among black veterans.^24^ They faced challenges from some staff who questioned the ethics of targeting hypertension resources specifically at the black veteran population, rather than at all veterans equally. This ethos of equality of care provision, over an overarching health equity goal, may be a barrier to allocating resources proportionate to need.

More generally, when there is a drive to address the wider determinants of health as part of QI planning (CMOC 3), and when implicit bias is tackled within institutions (CMOC 4), a culture may emerge to include equity considerations within QI. A willingness to address the wider determinants of health and knowledge of implicit bias both demonstrate how knowledge of health equity can be built into the culture of organisations' QI practices.^2426 27 34^,^38^

#### Resources

Resources refers to the practical and tangible elements that can impact equity considerations during QI projects. From the data, we identified two key subclusters related to resourcing: first, workload and staff capacity/capability, and second, multilingual resources. The first explores the material resources needed for effective QI. CMOCs 5 and 6 relate to staff workload and capacity to undertake QI with an equity focus. CMOC 5 details the time, training and resources that are needed to consider inequalities in QI. CMOC 6 describes the competing job responsibilities that may lead to staff having reduced capacity to undertake EFQI. For example, in a QI project focused on improving care gaps in early language delay, staff members reported competing job responsibilities as important in preventing adequate follow-up.^39^

The second subcluster relates to the provision of multilingual resources targeted at marginalised groups (CMOC 7A), along with sufficient training that encourages culturally competent care (CMOC 7B) to support minority ethnic and racial groups. Barceló *et al* undertook a QI project that aimed to decrease inequalities and improve mental health outcomes in black and Latino adults.^40^ Throughout the project materials were provided both in English and Spanish, and culturally competent care principles and resources were built into interventions. Outcomes improved for both groups, although according to different mental health-related measures, black adults experienced a reduced risk of poor mental health-related quality of life, while Latino adults experienced a greater probability of mental wellness.

When staff lack training, experience and exposure to the specific needs of groups with diverse health needs, they are more likely to undertake QI designed with the idea of an ‘average’ patient in mind. This patient may not be imagined as marginalised (CMOC 8). Cene *et al* undertook a QI project that explicitly aimed to reduce racial disparities in blood pressure management between African-American and white patients.^25^ The intervention was not more effective in African American patients than white patients. The authors suggested that this was because they did not ‘culturally tailor’ their intervention to the needs of African Americans.

#### Data

Both quantitative and qualitative data can alter how QI addresses inequalities. Cené *et al* used a mixed-methods approach in their QI intervention to reduce racial inequalities in hypertension in a rural primary care setting. Forty-one interviews with patients, providers and staff were used to inform the implementation phase of the study, and disaggregated quantitative data were in turn used to track the progress and efficacy of the intervention.^25^ The project had a sustained equity focus throughout, and was impactful on the health of the overall sample.

Furthermore, we found that using disaggregated data to identify problems and track improvements was central to facilitating QI that considers health inequalities throughout (CMOC 10A, 10B, 10C). The use of health equity dashboards or the production of other reports using disaggregated data can allow QI practitioners to stratify patients according to need, and understand pre-existing or emerging disparities in their target populations.^2326 27 32 35 37 39^,^43^

Conversely, the effective use of data can be hindered when accessing information from electronic patient records is difficult, or does not allow disaggregation (CMOCs 11A, 11B). Gagnon *et al* discuss the inaccessibility of patient records, and the limitations that electronic health record data capture can place on clinical care for LGBTQ+ patients. When electronic health record functionality was improved, staff members were able to meet their data-reporting aims, and to recognise more accurately the types of data important to good care missing from the record.^44^

#### Design

We identified four key CMOCs relating to QI design and inequalities. The first three (CMOCs 12, 13 and 14) explore *who* is involved in the design process. Data suggest that QI should engage with a broad range of patient groups to ensure that services are designed with the needs of these groups in mind (CMOC 12). Multiple documents emphasise that service user involvement and well-planned co-design were central to the success of projects.^3138 40 45^,^49^ Greenwood and Shiers outline that service user involvement in their project was ‘crucial’ to its success, helping to establish ‘an ethos for improvement; one of candour and collaboration’.^46^

Moreover, we observed the importance of multidisciplinary staff members in designing QI strategies. Multidisciplinary perspectives can ensure a comprehensive understanding of the complex needs of diverse patient groups is built into design (CMOC 13). We observed multidisciplinary perspectives contributing to positive staff perceptions of improvement initiatives,^48^ decreased non-attendance rates^34^ and more equitable clinical outcomes.^24 27 38 39 42^

When QI projects engage with a diverse range of patient groups and take on board multidisciplinary perspectives, this may lead to an ethos of co-creation being present (CMOC 14). Green *et al* describe how the thoughtful and consistent inclusion of service users working alongside a multidisciplinary team had a ‘significant impact’ on the solutions generated throughout the project, including the central project emphasis of involving patients with their own physical health as part of the intervention.^48^

Data suggest that if QI projects require patient engagement, effort and resources to be successful, there is a risk that they will increase inequalities by excluding those likely to have the highest/most complex needs. For example, Brown *et al* found that a QI initiative that offered patients same-day appointments increased inequalities in the postintervention period because of difficulties arranging transport at short notice.^39^

## Discussion

### Statement of principal findings

We found four key clusters of CMOCs that may lead to QI addressing or worsening health inequalities. First, the values and understanding that organisational leadership more broadly and QI practitioners individually hold: if there is pre-existing knowledge of health equity, QI is more likely to consider and potentially reduce inequalities. Second, the resources available to practitioners: if practitioners have capacity, manageable workloads and multilingual resources, QI is more likely to be equitable. Third, appropriate use of data: if practitioners can apply nuanced qualitative and quantitative data approaches, QI is more likely to highlight where inequities currently exist and aim to improve them. Finally, inclusive QI design can translate to more equitable outcomes: if QI is easy to use and designed in a patient-centric manner, it is more likely to be focused according to the needs of the patient population.

### Strengths and weaknesses of the study

This is the first realist review concerned with how QI can either increase, decrease or maintain health inequalities. The QI projects included looked at shorter-term outcomes, such as clinical or care outcomes, and we do not know the impact on long-term outcomes, for example, life expectancy. The majority of the studies were from North America and other areas of the Global North, meaning that specific types of inequality were disproportionately addressed; for example, a substantial proportion of the papers related to racial inequalities in the USA. Not all the CMOCs relating to racial inequalities may be transferable to other aspects of inequality, or to other national settings.

Identification of data was challenging. QI projects are often not published in peer-reviewed academic journals or are not classified as QI within published documents. As a result, we may have missed relevant articles. For example, we did not find any data relating to the impact of patient consent on data completeness or race-adjusted diagnostic protocols. There may also be systematic differences between QI projects that feature in the academic literature and those that do not.

### What the results mean

Our results demonstrate the importance of leadership and organisational culture. When organisational culture is committed to the equitable provision of healthcare, and reducing health inequalities more generally, QI projects appear more likely to consider inequalities. However, there is a lack of consensus of what health equity is, both conceptually and in practice. Multiple terms were used, such as disparities, inequalities and inequities. We used the National Health Service definition of health inequalities,^13^ acknowledging that multiple definitions exist.^50^ Compounding this, the QI approaches presented here vary from large analyses of electronic patient records to smaller qualitative projects. Projects use different QI methodologies and have varying definitions of quality itself.

A lack of time and resource were common challenges present in the data. These barriers are a problem for QI in general^51^ but become especially pertinent when attempting to do EFQI. Lack of resources—both financial and time—mean that QI projects are often designed without the patient groups they serve in mind, instead taking ‘the path of least resistance’. Previous research has proposed that highly ‘agentic’ public health interventions (those that require a high amount of personal resources, rather than those which address the structural determinants of health) are likely to widen inequalities between different groups.^52 53^ Our results echo these findings; QI interventions must be made easy for patients to benefit from if they are to be equitable for all. For example, a QI initiative aimed at long-term condition management that requires patients to book online, arrange transport and attend at a certain time will be harder for patients from disadvantaged backgrounds to benefit from. One way to ensure that all patients can benefit from QI is through careful planning and sustained engagement with different patient groups to better understand barriers to accessing care.

### Comparison with existing literature

Previous research^3^,^6^ supports many themes in our findings. Two commentaries informed much of our thinking when writing this review, and we consider this paper as contributing to a growing body of EFQI literature. Hirschhorn *et al* outline two of our four clusters in their call for EFQI, arguing that patient involvement and disaggregated data are central to realising QI that considers equity.^3^ Lion *et al* present four principles to introduce health equity approaches into QI work; disaggregated data, system-related factors, patient involvement, and understanding the context in which QI is undertaken.^4^

Both papers support our findings relating to the importance of including and consulting with a diverse patient group when undertaking QI. We extend this by drawing on previous research highlighting the utility of ‘democratic’ approaches to diverse and multidisciplinary service user and staff engagement.^20 54^ Similarly, both papers identify the importance of disaggregated data. Hirschorn *et al* describe this process as ‘designing better’ while Lion *et al* encourage QI practitioners to ensure ‘pre-existing disparities are well-understood’.^3 4^ Neither paper acknowledges the difficulties to implementation we have highlighted in CMOCs 10A–11B.

Importantly, neither paper highlights ‘resourcing’ or ‘values and understanding’ as central to EFQI. There is only implicit reference to the need for adequate resourcing to facilitate QI with an equity focus. For example, Lion *et al* acknowledge that language and literacy are ‘system-related’ issues and suggest that engagement activities should be structured with this in mind.^4^

### Implications for policy, practice and future research

QI activities across healthcare organisations have the potential to contribute to unfair and avoidable differences in health and care. To ensure that QI activities help address inequalities, policymakers and practitioners should focus on building organisational infrastructure to support EFQI. First, organisational leaders should build shared values and understanding of what inequalities exist, and allocate resources according to need. Second, managers should recognise that undertaking QI from an equity-focused perspective is not possible if staff do not have time and resources; ensuring dedicated time and space is key. Third, organisations should consider disaggregate quantitative data wherever possible, and develop qualitative data from the lived experience of disadvantaged groups and insights from multidisciplinary staff. Leveraging these data requires analytical support with interoperability. Finally, organisations should build long-term relationships with communities and patients, rather than attempting to establish new relationships for each project. Participatory approaches should be used to ensure that lived experiences and co-production are embedded in QI improvement initiatives.^55^

Future research should focus on the perspectives of healthcare staff and real-world examples of QI initiatives which address inequalities and take a systems approach. We do not believe that a new QI framework, in addition to the numerous existing frameworks, is required to promote EFQI, but rather efforts need to be made to optimise existing QI and make it more equitable.

## Conclusion

We found four key clusters (values and understanding, resources, data and design) that help to outline why, how, for whom and in which circumstances QI projects can impact inequalities in healthcare organisations. In the future, the way we think about EFQI needs to be multidisciplinary, inclusive, flexible and responsive to the needs of the most disadvantaged groups.

## Supplementary material

10.1136/bmjqs-2024-017386 online supplemental appendix 1

10.1136/bmjqs-2024-017386 online supplemental appendix 2

10.1136/bmjqs-2024-017386 online supplemental table 1

## Data Availability

Data are available upon reasonable request.
