# Supplementary material for: A realist review of how, why, for whom and in which contexts quality improvement in healthcare impacts inequalities
Source: BMJ Qual Saf. 2025 Jan 19;34(8):e017386. doi: 10.1136/bmjqs-2024-017386 (PMC12322391; doi:10.1136/bmjqs-2024-017386)
Supplement: online supplemental appendix 2 [file bmjqs-34-8-s002.pdf]

A realist review of how, why, for whom, and in which contexts quality improvement in health care impacts inequalities: CMOCs Document and Evidence Examples

Cluster One: Values and Understanding

CMOC Number	CMOC	Example data	Supporting papers
1A	When QI project developers are 'vocal' about the need for equity, and have knowledge about how inequity impacts health outcomes (C) then an ethical imperative for equity is embedded within QI projects (O) because they believe that equity constitutes a core part of QI (M)	**Burkitt et al., 2021:**<br>*Given that interventions targeting entire patient populations typically do not decrease racial disparities. it is recommended that efforts to close gaps in care first make a "concerted effort to achieve more rapid improvements among those who were worse off to start, within an overall strategy to improve everyone's health."*<br><br>*Viewing the issue from a distributive justice perspective, it is important to focus resources on improving BP control among the patients at greatest risk and are worst off at the start and should not be treating all patients the same*	Siegel *et al.*, 2012; Zhang *et al.*, 2014; Cykert *et al.*, 2020; Furness *et al.*, 2020; Burkitt *et al.*, 2021; Doran *et al.*, 2021; Davidson *et al.*, 2022
1B	When QI developers have an emotive and collective determination to address existing and historic racial injustice in healthcare (C) projects are designed and delivered to address these injustices (O) because there is an ethical imperative to do so (M)	**Cykert et al., 2020**<br>*When interpreting the results of ACCURE, some of the history concerning racial disparities must be considered. As noted by the Sullivan Commission (2004), the more visible racial barriers of the U.S. health care system were eradicated by the Civil Rights Era, but today's effects from "institutional racism" are subtle. Institutional racism has been defined as a process of oppression, unconscious or not, functioning as "a system of structuring opportunity and assigning value based on race phenotype, that unfairly disadvantages some and undermines the potential of the whole society". Examples include: lack of providers within reasonable traveling distance, poor institutional understanding of how to mobilize community organizations that principally serve Black residents, and racial discordance between*	Miranda *et al.*, 2003; Hassaballa *et al.*, 2015; Parker *et al.*, 2019; Cykert *et al.*, 2020; Main *et al.*, 2020; Davidson *et al.*, 2022; Gonzalez *et al.*, 2022; Meurer *et al.*, 2022

		patients and clinicians that may affect care-seeking behaviors. Our previous work demonstrating that Blacks with higher comorbid risk, poor perceptions of communication, or no regular source of care were less apt to receive cancer surgery served as a poignant example of how unintended, institutional biases can be operationalized.	
2	When staff concerns question the ethics around targeting QI interventions at specific disadvantaged groups rather than all groups who need healthcare (C) it may be more difficult to do EFQI (O) because they have concerns about pursuing the problem with an *equity* focus rather than an *equality* focus (M)	**Burkitt et al., 2021:**<br>Ethical concerns about targeting Black veterans with severe, uncontrolled hypertension rather than all veterans with severe hypertension.<br><br>*Despite the commitment of leadership to provide equitable care in our initiative, facility-level leaders and providers questioned the ethics of focusing QI efforts on Black veterans with severe hypertension, rather than all patients with severe hypertension or even all patients with uncontrolled blood pressure.*	Jean-Jacques *et al.*, 2012; Cykert *et al.*, 2020; Burkitt *et al.*, 2021
3	When staff developing QI have a collective understanding of the wider determinants of health (C) they are more likely to design QI projects with fewer barriers that prevent disadvantaged patients from accessing them (O) because they have a commitment to social medicine	**Meurer et al., 2022:**<br>*Further research, advocacy and continuing education will improve the ability of pediatric professionals to form effective partnerships with community agencies to address the social determinants of health when caring for children who live in poverty. Principles and strategies have been identified to guide policy, practice and advocacy to help infants, toddlers and families at higher risk for poor outcomes. Both high quality medical care and community policies and systems to address social determinants of health are needed to ensure child well-being.*	Doherty *et al.*, 2016; Burkitt *et al.*, 2021; Meurer *et al.*, 2022; Steinbock *et al.*, 2022
4	When QI staff work in an organisation that makes efforts to address implicit bias (C) then they are more likely to create EFQI projects (O) because they are more aware of their own implicit biases, and how these biases might contribute to the 'root causes' of health disparity (M)	**Cykert et al., 2020:**<br>*Health equity training sessions covering concepts such as implicit bias, gatekeeping, and institutional racism were offered to all staff and providers quarterly to make them aware of these concepts*	Leeds *et al.*, 2017; Cykert *et al.*, 2020; Davidson *et al.*, 2022; Matthews *et al.*, 2022

Cluster Two: Resources

CMOC Number	CMOC	Example	Supporting papers
5	When staff are given the time, training and resources to address inequalities in projects (C) they are more likely to consider the question of health equity (O) because they feel enabled to do so (M)	**Gagnon et al., (2022):** *Staff training provided specific information and education focused on the work of the initiative, and/ or integrated this information into pre-existing training opportunities like employee orientation. Participants cited these trainings as having a positive impact on both processes and outcomes related to the initiative, as they increased awareness and understanding of the ongoing work.*	Miranda *et al.*, 2003; Ngo *et al.*, 2009; 2010; Olomu *et al.*, 2010; Siegel *et al.*, 2012; Badrick *et al.*, 2014; Doherty *et al.*, 2016; Greenwood and Shiers, 2016; Watanabe *et al.*, 2016; Green *et al.*, 2018; Harrold *et al.*, 2018; Barceló *et al.*, 2019; Parker *et al.*, 2019; Cykert *et al.*, 2020; Furness *et al.*, 2020; Gallaher *et al.*, 2020; Main *et al.*, 2020; Burkitt *et al.*, 2021; Doran *et al.*, 2021; Davidson *et al.*, 2022; Gagnon *et al.*, 2022; Gonzalez *et al.*, 2022; Matthews *et al.*, 2022; Meurer *et al.*, 2022; Steinbock *et al.*, 2022; Behling *et al.*, 2023
6	When staff tasked with time consuming QI activity are also experiencing high workloads and administrative responsibilities (C) QI occurs via the most expeditious means (O) because staff have reduced capacity and/or bandwith to engage with projects (M)	**Brown et al., (2016):** *After January 2013, when the task of follow-up phone calls was transitioned to a temporary staff member, the staff member reported some calls were delayed or missed due to competing job responsibilities.*	Brown *et al.*, 2016; Greenwood and Shiers, 2016; Green *et al.*, 2018; Harrold *et al.*, 2018; Gallaher *et al.*, 2020; Martinez *et al.*, 2021; Gagnon *et al.*, 2022
7A	When minority ethnic patients are provided with high quality, multilingual, culturally competent resources and services (C) they are	**Parker et al., (2019):** *Regarding racial and ethnic disparities, given limited racial and ethnic differences in process and outcome measures in year 1, we sought to determine more targeted interventions in years 2 and 3. We conducted*	Miranda *et al.*, 2003; Ngo *et al.*, 2009; Olsson *et al.*, 2014; Watanabe *et al.*, 2016; Parker *et al.*, 2019; Martinez *et al.*, 2021

	more likely to feel confident about the service (O) because they feel respected and supported (M)	*in-depth qualitative interviews with non-Hispanic black and Hispanic mothers at 2 hospitals to better understand facilitators and barriers to prolonged lactation. Relevant facilitators included perceptions of strong practical and emotional support by NICU staff when delivered in a racially and ethnically unbiased manner in the mother's primary language.*<br><br>*The leadership team created multilingual family education materials that were released in 2016 and videos that were released in 2018.*	
7B	When staff are provided with adequate training to serve minority ethic patients (C) they are more likely to provide high quality, equitable care (O) because they feel confident (M)	**See above**	See above
8	When staff have a lack of training, experience and exposure to the specific needs of diverse groups with different health needs (C) QI is designed and delivered for a preconceived idea of what the 'average' patient needs from a service (O) because staff lack awareness of the need to design and deliver EFQI (M)	**Cene et al., (2017)**<br>*We propose two possible explanations for why our intervention was not differentially more effective in AAs than whites. First, our intervention was a practice-level QI intervention that employed strategies that have been associated with BP improvement in racial minority populations, such as supported self-management, home BP monitoring, continuing medical education, practice facilitation, and team- based HTN care; however, we did not culturally tailor our intervention to AAs.*	Al-Khatib *et al.*, 2012; Badrick *et al.*, 2014; Greenwood and Shiers, 2016; Cené *et al.*, 2017; Furness *et al.*, 2020

Cluster Three: Data

CMOC Number	CMOC	Example data	Supporting papers
9	When organisations running QI have a culture of listening to, collating, analysing and responding and reacting to the experiences of diverse staff and patients about the project (C) they are more likely to have a better understanding of the project's impact (O) because of the wide range of knowledge gained (M)	**Greenwood and Shiers (2016):** *As the local improvement programmes went forward participants were encouraged to note what "worked well/what was difficult" to inform a qualitative appreciation of the implementation. This was fed back to participating sites on a continuing basis, to encourage shared learning and development in preparation for the final phase of re-audit.*	Olsson *et al.*, 2014; Hassaballa *et al.*, 2015; Brown *et al.*, 2016; Greenwood and Shiers, 2016; Cené *et al.*, 2017; Parker *et al.*, 2019; Burkitt *et al.*, 2021; Gagnon *et al.*, 2022; Meurer *et al.*, 2022
10A	When the developers of QI projects have access to disaggregated granular data that enables them to identify the needs of specific people and communities (C) then they are more able to ensure that EFQI is being undertaken (O) because they have a better understanding of need in the population served (M)	**Badrick et al., 2014** *Identifying differences in chronic disease management by age, ethnicity and gender is the first step to developing and implementing strategies to reduce some of the observed inequalities. Reports were received positively by practices, which were keen to reflect on their data and consider strategies for change; this process was aided by facilitation.*	Al-Khatib *et al.*, 2012; Siegel *et al.*, 2012; Badrick *et al.*, 2014; Zhang *et al.*, 2014; Brown *et al.*, 2016; Cené *et al.*, 2017; Leeds *et al.*, 2017; Barceló *et al.*, 2019; Cykert *et al.*, 2020; Davidson *et al.*, 2022; Meurer *et al.*, 2022; Behling *et al.*, 2023
10B	When the developers of QI projects have access to disaggregated granular data that enables them to monitor the progress of an EFQI project (C) then they are more able to ensure that EFQI is being undertaken (O) because they have a better understanding of the project's impacts (M)	**Davidson et al., 2022:**<br><br>*We continued to present stratified SMM (severe maternal morbidity) and SMM- H (severe maternal morbidity from hemorrhage) data at monthly department meetings and incorporated discussions on national disparities in maternal morbidity and mortality and potential root causes, including implicit bias and lack of standardised clinical care.*	See above
10C	When the developers of QI projects have access to disaggregated granular data that enables them to identify impacts on specific people (C) then they are more able to assess the health	**Cykert et al., (2020):**<br><br>*In this vain, all consented patients received the intervention which consisted of a real time warning system derived from automated uploads of data from electronic health records (EHRs) to enhance*	See above

	equity impact of the project itself (O) because they have a more holistic understanding of impact (M)	*transparency, feedback to clinical teams on completion of cancer treatments according to race (enhance both transparency and accountability), and a nurse navigator with access to the warning system (to formalize accountability).*	
11A	When patient electronic records are incomplete (C) then EFQI is more difficult to carry out (O) because staff members struggle to find the correct patient data and to contact the patients most in need (M)	**Gonzalez et al., 2022:**<br>*A secondary area of feedback was the inaccuracy of patient eligibility. A substantial portion of the initial patients that were called reported already having received the vaccine despite no record of this in the EHR (electronic health record). This was due to multiple places in the EHR to log patient vaccination status as well as delays in the migration of vaccination data into the EHR from other databases.*	Brown *et al.*, 2016; Greenwood and Shiers, 2016; Gallaher *et al.*, 2020; Gagnon *et al.*, 2022; Gonzalez *et al.*, 2022
11B	When patient electronic records are difficult to combine and analyse (C) then EFQI is more difficult to carry out (O) because staff members have difficulty finding which individuals and patient groups more broadly are in need of specific services (M)	**Gagnon et al., 2022:**<br>*A lack of timely EHR (electronic health record) vendor or internal information technology support made it challenging to incorporate SOGI (sexual orientation and gender identity) questions and data collection fields into the EHR. Doing so required FQHCs to create new fields in their EHR systems or purchase additional applications from their EHR vendor. FQHCs (federally qualified health centres) that were able to input SOGI data into their EHR often had difficulty extracting the data for clinical use and analysis. This challenge was described in the following exchange during an interview:*<br>*QI Facilitator Site 1: We still have a little bit of a struggle with the data, too, because our EHR system can be a little cumbersome when it comes to data*	See above

Cluster Four: Design

CMOC Number	CMOC	Example data	Supporting Papers
12	When QI projects have and incorporate considerate representation of communities in which service user perspectives are given equal credence (C) QI projects will have a shared creation of knowledge, goals and services that are grounded in the lived experience of the target community (O) because they take on board the perspective of the community (M)	**Green et al., (2018)** *Service user involvement within the initiative was critical to the success through the development of the patient-held record and in establishing an ethos for improvement: one of candour and collaboration. The 4PI framework, developed for involving patients, was supportive in creating an improvement team with flattened hierarchies and set a tone for inclusivity and a culture of shared learning emphasising that all contributions are of equal value. However, the team reflected that it was the active involvement of service users and front line clinical staff in co-producing the interventions that was generally seen as significant added value to the initiative, as involving those delivering or using services was a key aspect*	Olsson *et al.*, 2014; Greenwood and Shiers, 2016; Watanabe *et al.*, 2016; Green *et al.*, 2018; Barceló *et al.*, 2019; Parker *et al.*, 2019; Furness *et al.*, 2020; Matthews *et al.*, 2022
13	When QI projects involve multidisciplinary staff as equal partners in the design process (C) then QI initiatives will take a broad and inclusive approach (O) because a rich understanding of the multidimensional needs of patients is included in design (M)	**Green et al., (2018):**<br><br>*The multi-professional improvement team was established by a senior psychologist and psychiatrist, the co-leads for the initiative, to include staff from nursing, pharmacy, therapies staff including a fitness trainer, senior management, service user representatives and QI. Involving those most likely to be affected by any changes was necessary to ensure both the acceptability of any change made and allow all key stakeholders to contribute to the project through the design and testing of interventions.*	Ngo *et al.*, 2009; Siegel *et al.*, 2012; Hassaballa *et al.*, 2015; Brown *et al.*, 2016; Doherty *et al.*, 2016; Green *et al.*, 2018; Barceló *et al.*, 2019; Parker *et al.*, 2019; Furness *et al.*, 2020; Gallaher *et al.*, 2020; Burkitt *et al.*, 2021; Doran *et al.*, 2021; Martinez *et al.*, 2021; Satti *et al.*, 2021; Davidson *et al.*, 2022; Gagnon *et al.*, 2022; Matthews *et al.*, 2022; Meurer *et al.*, 2022
14	When there is an ethos of co-creation embedded within the hospital and target patient groups and representations are deeply involved (C) then QI projects are more likely to	**Gagnon et al., 2022** *FQHCs (federally qualified health centres) were also able to hire additional staff (ie, outreach coordinators and pre- exposure prophylaxis (PrEP) Navigators) and conduct appropriate referrals to community agencies focused on quality of care for SGM patients. Community*	Olsson *et al.*, 2014; Greenwood and Shiers, 2016; Green *et al.*, 2018; Gagnon *et al.*, 2022

	have an equity focus (O) because mutual respect means that the voices of the target group are more likely to be heard (M)	*partnerships emerged as a key facilitator to overall capacity to address healthcare disparities for SGM patients. Ultimately, these partnerships were a facilitator not only to FQHCs' ability to provide care, but also to their ability to develop more trusting relationships within the SGM community at large*	
15	When QI initiatives are designed such that they require a lot of patient agency, effort and engagement in order to gain the benefits (C) then those with the highest need and most significant challenges are the least likely to benefit from QI (O) because they most often lack the resources needed to engage with the initiative (M)	**Brown et al., 2016:**<br>*The process for scheduling appointments was not well explained to parents, leading to difficulties navigating the health care system.*	Olsson *et al.*, 2014; Zhang *et al.*, 2014; Berkowitz *et al.*, 2015; Hassaballa *et al.*, 2015; Brown *et al.*, 2016; Parker *et al.*, 2019; Burkitt *et al.*, 2021; Gonzalez *et al.*, 2022; Meurer *et al.*, 2022

Al-Khatib, S.M. *et al.* (2012) ‘Trends in Use of Implantable Cardioverter-Defibrillator Therapy Among Patients Hospitalized for Heart Failure’, *Circulation*, 125(9), pp. 1094–1101. Available at: https://doi.org/10.1161/CIRCULATIONAHA.111.066605.

Badrick, E. *et al.* (2014) ‘Health equity audits in general practice: a strategy to reduce health inequalities’, *Primary Health Care Research & Development*, 15(1), pp. 80–95. Available at: https://doi.org/10.1017/S1463423612000606.

Barceló, N.E. *et al.* (2019) ‘Community Engagement and Planning versus Resources for Services for Implementing Depression Quality Improvement: Exploratory Analysis for Black and Latino Adults’, *Ethnicity & Disease*, 29(2), pp. 277–286. Available at: https://doi.org/10.18865/ed.29.2.277.

Behling, E.M. *et al.* (2023) ‘Improvement in Hypertension Control Among Adults Seen in Federally Qualified Health Center Clinics in the Stroke Belt: Implementing a Program with a Dashboard and Process Metrics’, *Health Equity*, 7(1), pp. 89–99. Available at: https://doi.org/10.1089/heq.2022.0109.

Berkowitz, S.A. *et al.* (2015) ‘Building Equity Improvement into Quality Improvement: Reducing Socioeconomic Disparities in Colorectal Cancer Screening as Part of Population Health Management’, *Journal of General Internal Medicine*, 30(7), pp. 942–949. Available at: https://doi.org/10.1007/s11606-015-3227-4.

Bhalla, R. *et al.* (2010) ‘Improving Primary Percutaneous Coronary Intervention Performance in an Urban Minority Population Using a Quality Improvement Approach’, *American Journal of Medical Quality*, 25(5), pp. 370–377. Available at: https://doi.org/10.1177/1062860610367958.

Brown, C.M. *et al.* (2016) ‘Narrowing Care Gaps for Early Language Delay’, *Clinical Pediatrics*, 55(2), pp. 137–144. Available at: https://doi.org/10.1177/0009922815587090.

Burkitt, K.H. *et al.* (2021) ‘Evaluation of a collaborative VA network initiative to reduce racial disparities in blood pressure control among veterans with severe hypertension’, *Healthcare*, 8, p. 100485. Available at: https://doi.org/10.1016/j.hjdsi.2020.100485.

Cené, C.W. *et al.* (2017) ‘A multicomponent quality improvement intervention to improve blood pressure and reduce racial disparities in rural primary care practices’, *The Journal of Clinical Hypertension*, 19(4), pp. 351–360. Available at: https://doi.org/10.1111/jch.12944.

Cykert, S. *et al.* (2020) ‘A Multi-faceted Intervention Aimed at Black-White Disparities in the Treatment of Early Stage Cancers: The ACCURE Pragmatic Quality Improvement trial’, *Journal of the National Medical Association*, 112(5), pp. 468–477. Available at: https://doi.org/10.1016/j.jnma.2019.03.001.

Davidson, C. *et al.* (2022) ‘Examining the effect of quality improvement initiatives on decreasing racial disparities in maternal morbidity’, *BMJ Quality & Safety*, 31(9), pp. 670–678. Available at: https://doi.org/10.1136/bmjqs-2021-014225.

Doherty, A.M. *et al.* (2016) ‘Improving quality of diabetes care by integrating psychological and social care for poorly controlled diabetes: 3 Dimensions of Care for Diabetes’, *International journal of psychiatry in medicine*, 51(1), pp. 3–15. Available at: https://doi.org/10.1177/0091217415621040.

Doran, E. *et al.* (2021) ‘Improving access to epilepsy care for homeless patients in the Dublin Inner City: a collaborative quality improvement project joining hospital and community care’, *BMJ Open Quality*, 10(2), p. e001367. Available at: https://doi.org/10.1136/bmjoq-2021-001367.

Furness, B.W. *et al.* (2020) ‘Transforming Primary Care for Lesbian, Gay, Bisexual, and Transgender People: A Collaborative Quality Improvement Initiative’, *The Annals of Family Medicine*, 18(4), pp. 292–302. Available at: https://doi.org/10.1370/afm.2542.

Gagnon, K.W. *et al.* (2022) ‘Qualitative inquiry into barriers and facilitators to transforming primary care for lesbian, gay, bisexual and transgender people in US federally qualified health centres’, *BMJ Open*, 12(2), p. e055884. Available at: https://doi.org/10.1136/bmjopen-2021-055884.

Gallaher, C. *et al.* (2020) ‘The St Thomas’ Hospital Emergency Department Homeless Health Initiative: improving the quality, safety and equity of healthcare provided for homeless patients attending the ED’, *BMJ Open Quality*, 9(1), p. e000820. Available at: https://doi.org/10.1136/bmjoq-2019-000820.

Gonzalez, C.J. *et al.* (2022) ‘Development of a Practice-based Community Outreach Intervention to Prevent Inequities in COVID-19 Vaccinations’, *American Journal of Medical Quality*, 37(4), p. 348. Available at: https://doi.org/10.1097/JMQ.0000000000000049.

Green, S. *et al.* (2018) ‘Implementing guidelines on physical health in the acute mental health setting: a quality improvement approach’, *International Journal of Mental Health Systems*, 12(1), p. 1. Available at: https://doi.org/10.1186/s13033-018-0179-1.

Greenwood, P.J. and Shiers, D.E. (2016) ‘Don’t just screen intervene; a quality improvement initiative to improve physical health screening of young people experiencing severe mental illness’, *Mental Health Review Journal*, 21(1), pp. 48–60. Available at: https://doi.org/10.1108/MHRJ-01-2015-0003.

Harrold, S.A. *et al.* (2018) ‘Increasing physical activity for veterans in the Mental Health Intensive Case Management Program: A community-based intervention’, *Perspectives in Psychiatric Care*, 54(2), pp. 266–273. Available at: https://doi.org/10.1111/ppc.12233.

Hassaballa, H. *et al.* (2015) ‘Evaluation of a Diabetes Care Coordination Program for African-American Women Living in Public Housing’, *Journal of Clinical Outcomes Management*, 22(8). Available at: https://www.mdedge.com/jcomjournal/article/146525/diabetes/evaluation-diabetes-care-coordination-program-african-american (Accessed: 24 May 2023).

Jean-Jacques, M. *et al.* (2011) ‘Changes in Disparities Following the Implementation of a Health Information Technology-Supported Quality Improvement Initiative’, *Journal of General Internal Medicine*, 27(1), pp. 71–77. Available at: https://doi.org/10.1007/s11606-011-1842-2.

Leeds, I.L. *et al.* (2017) ‘Racial and Socioeconomic Differences Manifest in Process Measure Adherence for Enhanced Recovery After Surgery Pathway’, *Diseases of the colon and rectum*, 60(10), pp. 1092–1101. Available at: https://doi.org/10.1097/DCR.0000000000000879.

Main, E.K. *et al.* (2020) ‘Reduction in racial disparities in severe maternal morbidity from hemorrhage in a large-scale quality improvement collaborative’, *American Journal of Obstetrics and Gynecology*, 223(1), p. 123.e1-123.e14. Available at: https://doi.org/10.1016/j.ajog.2020.01.026.

Martinez, E.M. *et al.* (2021) ‘Improving Equity of Care for Patients with Limited English Proficiency Using Quality Improvement Methodology’, *Pediatric Quality & Safety*, 6(6), p. e486. Available at: https://doi.org/10.1097/pq9.0000000000000486.

Matthews, K.C. *et al.* (2022) ‘Enhanced Recovery after Surgery for Cesarean Delivery: A Quality Improvement Initiative’, *American Journal of Perinatology*, p. s-0042-1754405. Available at: https://doi.org/10.1055/s-0042-1754405.

Meurer, J. *et al.* (2022) ‘Improving Child Development Screening: Implications for Professional Practice and Patient Equity’, *Journal of Primary Care & Community Health*, 13, p. 21501319211062676. Available at: https://doi.org/10.1177/21501319211062676.

Miranda, J. *et al.* (2003) ‘Improving Care for Minorities: Can Quality Improvement Interventions Improve Care and Outcomes For Depressed Minorities? Results of a Randomized, Controlled Trial’, *Health Services Research*, 38(2), pp. 613–630. Available at: https://doi.org/10.1111/1475-6773.00136.

Ngo, V.K. *et al.* (2009) ‘Outcomes for Youths From Racial-Ethnic Minority Groups in a Quality Improvement Intervention for Depression Treatment’, *Psychiatric Services*, 60(10), pp. 1357–1364. Available at: https://doi.org/10.1176/ps.2009.60.10.1357.

Olomu, A.B. *et al.* (2010) ‘Evidence of disparity in the application of quality improvement efforts for the treatment of acute myocardial infarction: The American College of Cardiology’s Guidelines Applied in Practice Initiative in Michigan’, *American Heart Journal*, 159(3), pp. 377–384. Available at: https://doi.org/10.1016/j.ahj.2009.12.014.

Olsson, E. *et al.* (2014) ‘Community collaboration to increase foreign-born women´s participation in a cervical cancer screening program in Sweden: a quality improvement project’, *International Journal for Equity in Health*, 13(1), p. 62. Available at: https://doi.org/10.1186/s12939-014-0062-x.

Parker, M.G. *et al.* (2019) ‘Addressing Disparities in Mother’s Milk for VLBW Infants Through Statewide Quality Improvement’, *Pediatrics*, 144(1), p. e20183809. Available at: https://doi.org/10.1542/peds.2018-3809.

Poots, A.J. *et al.* (2014) ‘Improving mental health outcomes: achieving equity through quality improvement’, *International Journal for Quality in Health Care*, 26(2), pp. 198–204. Available at: https://doi.org/10.1093/intqhc/mzu005.

Satti, K.F. *et al.* (2021) ‘Improving Care for Childhood Obesity: A Quality Improvement Initiative’, *Pediatric Quality & Safety*, 6(3), p. e412. Available at: https://doi.org/10.1097/pq9.0000000000000412.

Sequist, T.D. *et al.* (2006) ‘Effect of Quality Improvement on Racial Disparities in Diabetes Care’, *Archives of Internal Medicine*, 166(6), pp. 675–681. Available at: https://doi.org/10.1001/archinte.166.6.675.

Siegel, B. *et al.* (2012) ‘A Quality Improvement Framework for Equity in Cardiovascular Care: Results of a National Collaborative’, *Journal for Healthcare Quality*, 34(2), pp. 32–43. Available at: https://doi.org/10.1111/j.1945-1474.2011.00196.x.

Steinbock, C.M. *et al.* (2022) ‘Reducing Disparities: A Virtual Quality Improvement Collaborative Resulted in Better Health Outcomes for 4 Target Populations Disproportionately Affected by HIV’, *Journal of Public Health Management and Practice*, 28(2), pp. 162–169. Available at: https://doi.org/10.1097/PHH.0000000000001360.

Watanabe, M.K. *et al.* (2016) ‘The Impact of Risk-Based Care on Early Childhood and Youth Populations’, *Journal of the California Dental Association*, 44(6), pp. 367–377. Available at: https://doi.org/10.1080/19424396.2016.12221025.

Zhang, R. *et al.* (2014) ‘Factors Influencing the Increasing Disparity in LDL Cholesterol Control Between White and Black Patients With Diabetes in a Context of Active Quality Improvement’, *American Journal of Medical Quality*, 29(4), pp. 308–314. Available at: https://doi.org/10.1177/1062860613498112.
