# Supplementary material for: A realist review of how, why, for whom and in which contexts quality improvement in healthcare impacts inequalities
Source: BMJ Qual Saf. 2025 Jan 19;34(8):e017386. doi: 10.1136/bmjqs-2024-017386 (PMC12322391; doi:10.1136/bmjqs-2024-017386)
Supplement: online supplemental table 1 [file bmjqs-34-8-s003.pdf]

Document Characteristics

Supplementary Table 1: Gap Interventions

| Author and Year | Country | Target PROGRESS+ Group | Setting, health condition and number of participants | Methods/ Type of data | Aim | Outcome |
|---|---|---|---|---|---|---|
| Al-Khatib et al., 2012<br>(44) | USA | Race, gender | Secondary care – hospital/ Heart failure/ 11880 participants | Statistical analysis of those enrolled in GWTG QI programme | To reduce sex and racial disparities | **No difference in inequalities.** ICD use increased in overall study population between 2005-2007 (p= 0.0008) and in all race and sex groups; black women (p=0.0008), white women (p=0.010), black men (p=0.0009) and white men (p= 0.0072). Compared with white men, black and white women had a lower probability of ICD use after the intervention. Inequalities improved in ethnic groups but persisted across male and female sex groups. |
| Badrick et al., 2014<br>(46) | UK | Age, gender, ethnicity | Primary care/ Coronary heart disease, T2 diabetes, COPD/ 800000+ participants | Health equity audit | To reduce disparities through data visibility | **No difference in inequalities.** All ethnic groups showed improvement in the four outcome measures included, but a reduction in inequalities between ethnic groups did not occur. |
| Barceló et al., 2019<br>(43) | USA | Race | Community care/ Depression/ 1018 participants | RCT to assess efficacy of CEP (Community | To reduce racial disparities in depression care | **Narrowing inequalities.** CEP reduced the odds of poor mental health related quality of life for black adults (p=0.028) and resulted in a |

| | | | | | | |
|---|---|---|---|---|---|---|
| | | | | Engagement and Planning) | | greater probability for mental wellness for Latino adults (p=0.034). |
| Behling et al., 2023 (26) | USA | Race | Primary care/ Hypertension/ 45948 participants | Statistical analysis of those enrolled in the AMA MAP BP QI programme | To reduce racial disparities in hypertension | **No difference in inequalities.** After the intervention, a decline in systolic BP after adding anti-hypertensive medication and adequate 30 day follow up was less in non-Hispanic black vs non-Hispanic white adults (p=<0.001). |
| Berkowitz et al., 2015 (61) | USA | Socio-economic status | Primary care/ Colorectal cancer/ 51442 (18 primary care practice sites) | Quasi-experimental time-series analysis | To reduce disparities by SES in colorectal cancer screening rates | **Narrowing inequalities.** Overall screening in the population increased, and inequalities in colorectal screening by educational attainment group 'moderately decreased', attributable to the TopCare intervention. |
| Brown et al., 2016 (42) | USA | Age, socio-economic status | Primary care/ Speech delay/ 470 referrals | Mixed methods | To increase the percentage of children 0 to 3 years referred to the service attending initial appointments from 40-60% | **Narrowing inequalities.** Inequalities were eliminated post-intervention for families from low-income neighbourhoods. After the intervention there was no longer a significant difference in adherence rates (44% vs 53% p=0.15). |
| Burkitt et al., 2021 (27) | USA | Race | Secondary care -veterans affairs medical centre/ Hypetension/ 9913 participants | Comparative statistics and regression modelling | To decrease racial disparities in BP control | **Narrowing inequalities.** The proportion of black veterans with severe hypertension decreased significantly (p=0.002) and racial disparity in severe hypertension decreased by –0.9% (p=0.01). |

| | | | | | | |
|---|---|---|---|---|---|---|
| Cené et al., 2017<br>(28) | USA | Race | Primary care/ Hypertension/ 525 referrals | Non-randomised observational trial with qualitative elements | To improve BP control and assess changes by race | **No difference in inequalities.** No significant racial disparity was shown at baseline, and the intervention lowered mean SBP in both African Americans and Whites. There was no differential effect by race. |
| Cykert et al., 2019<br>(29) | USA | Race | Secondary care – cancer centre/ Lung and Breast cancer/ 302 referrals | RCT | To decrease racial disparities in cancer outcomes | **Narrowing inequalities.** Intervention reduced inequalities in treatment completion by race. Treatment completion in the control (79.7% for blacks and 87.3% for whites) and retrospective (83.1% for blacks and 90.1% for whites) groups showed significant inequalities. Black patients in the intervention group achieved a treatment completion rate of 88.4% compared to 89.5% for white patients. However, these results were not significant (p=0.77).. |
| Davidson et al., 2022<br>(30) | USA | Race, gender | Secondary care – hospital/ Maternal morbidity/ 13,659 deliveries | Descriptive statistics | To decrease racial disparities in severe maternal mortality by haemorrhage (SSM-H) | **Narrowing inequalities.** After the intervention the rate of SSM-H in Black women decreased from 45.5% to 31.6%. The rates for non-Hispanic white women and non-Hispanic Asian women were 25.7% and 24.5% respectively. Post intervention this disparity was no longer significant (p=0.0138). |
| Jean-Jaques et al., 2011<br>(62) | USA | Race, gender | Secondary care/ Coronary heart disease, heart failure, hypertension, diabetes/ 8919 participants | Descriptive statistics and regression modelling | To examine the effects of a health information technology intervention on disparities | **Mixed effects on inequalities**. Care quality improved for 14/7 measures for white patients and 10/17 measures for black patients. 7 measures had racial disparities at baseline – out of these, disparities declined for 2 , |

| | | | | | | |
|---|---|---|---|---|---|---|
| | | | | | | remained stable for 4 and increased for 1 measure. |
| Leeds et al., 2017 (40) | USA | Ethnicity, socio-economic status | Tertiary academic medical centre/ Surgical resection/ 639 participants | Descriptive statistics | To examine the effects of an ERAS (enhanced recovery after surgery)pathway on disparities | **No difference in inequalities.** ERAS improved length of stay in all sub-groups. Overall adherence to process measures was 31.7% in white patients and 26.5% in black patients (p=0.32). Low SES patients (17.1%) were less likely to be adherent than high SES patients (31.8%) (p=0.05). Epidural use was less in non-white patients (44.1% versus 57.1%, p = 0.02). |
| Main et al., 2020 (31) | USA | Race, gender | Secondary care/ Maternal morbidity / 73476 participants | Descriptive statistics and Poisson models | To examine the effect of a QI collaborative on racial disparities in severe maternal mortality by haemorrhage (SSM-H) | **Narrowing inequalities.** Pre-intervention disparities in SSM-H existed between black (28.6%) and white (19.8%) women. All mothers benefited from the intervention, but black mothers benefited more (9% absolute rate reduction for black mothers vs. 2.1 absolute rate reduction for white mothers). The black-white relative risk of severe maternal morbidity was 1.22 in the pre-intervention period and narrowed to 1.07 in the post-intervention period. |
| Matthews et al., 2022 (41) | USA | Race, gender | Secondary care/ C-section/ 1729 participants | Descriptive statistics | To evaluate an ERAS pathway on reducing length of hospital stay | **Narrowing inequalities.** Pre-intervention there were inequalities in length of stay between white patients and patients of all other ethnic |

| | | | | | | |
|---|---|---|---|---|---|---|
| | | | | | | groups. Post-intervention, this disparity was no longer seen |
| Meurer et al., 2022<br>(38) | USA | Race, socio-economic status | Primary care/ Child developmnetal screening/ 30000+ participants | Mixed methods | To improve child developmental screening | **No difference in inequalities.** Overall screening compliance improved. However compliance rates were significantly lower when examined by race/ethnicity, insurance status and income. |
| Miranda et al., 2003<br>(32) | USA | Race | Primary care/ Depression/ 778 participants | RCT | To reduce disparities in depression care and outcomes by ethnic group | **Mixed effects on inequalities.** The intervention improved care for all ethnic groups. The intervention decreased the likelihood that Latinos and African Americans would report probable depression at 6 and 12 months. The white intervention sample showed no difference from the control group. The intervention increased likelihood of employment for whites but not for other ethnic groups. Overall minority groups were more likely to be depressed and less likely to receive appropriate care even after the intervention. |
| Ngo et al., 2009<br>(33) | USA | Race | Primary care/ Depression/ 325 participants | RCT | See above. | See above. |

| | | | | | | |
|---|---|---|---|---|---|---|
| Olomu et al., (2010) (63) | USA | Race | Secondary care/ Cardiovascular health/ 2367 participants | Descriptive statistics and post-hoc analysis | To evaluate if a structured QI initiative could improve care for patients with acute myocardial infarction and decrease inequalities | **Widening inequalities.** Post intervention, non-white patients were 28% less likely to have had the GAP discharge tool used (p=0.004) and to receive smoking cessation counselling (p=<0.001). |
| Parker et al. 2019 (34) | USA | Race | Secondary care/ Breastfeeding/ 1670 participants | Mixed methods | To reduce racial and ethnic disparities in the provision of mother's milk | **Widening inequalities.** Disparities emerged at the 3 week mark post-intervention; at discharge 69% of white mothers, 53% of black mothers, and 48% of Hispanic mothers provided milk (P < .001) |
| Siegel et al., 2012 (45) | USA | Race | Secondary care/ Cardiovascular health/ 10 hospital sites included | Comparative statistics (chi squared tests) used to compare hospital performance | To improve heart failure care given to Black and Hispanic patients | **Narrowing inequalities.** Health inequalities were eliminated across all 10 participating hospitals by the end of the intervention period. |
| Steinbock et al., 2022 (39) | USA | Race, gender, age | Secondary care/ HIV/AIDS/ 19442 participants | Statistical analysis – regression modelling | To improve viral suppression rates across groups at risk of HIV/AIDS | **Narrowing inequalities.** Improvements in the gap in viral suppression rates were found across all target populations. The largest reduction in health inequalities was seen in transgender populations. |
| Zhang et al., 2015 | USA | Race | Primary care/ Cholesterol level/ 962 participants | Descriptive statistics and | Improve performance for multiple chronic | **Widening inequalities.** Inequalities increased by race after the intervention. There was a 12.1% increase in inequality between 2008 and |

| | | | | | | |
|---|---|---|---|---|---|---|
| (35) | | | | electronic health record review | disease and preventative care measures | 2010. Black patients were significantly less likely to have any LLD medication on their medication list both pre and post intervention (P=<0.001) |

Supplementary Table 2: Targeted Interventions

| Author and Year | Country | Target PROGRESS+ Group | Setting, health condition and number of participants | Methods/ Type of data | Aim | Outcome |
|---|---|---|---|---|---|---|
| Doherty et al., 2016 (37) | UK | Socio-economic status, race | Secondary care/ Diabetes/ 119 referrals | Mixed methods | To improve glycaemic control, reduce psychological distress and improve social functioning | **Generally positive results.** Non-attendance at appointments dropped. |
| Doran et al., 2021 (64) | Ireland | Homeless populations | Primary and secondary care/ Epilepsy/ 46 participants | Mixed methods | To use a multistakeholder co-production approach to design a new pathway of care for homeless patients | **Generally positive results.** Improvements to services and the development of new service pathways were recorded. |

| | | | | | | |
|---|---|---|---|---|---|---|
| Furness et al., 2020<br>(52)<br>And<br>Gagnon et al., 2022<br>(47) | USA | LGBT populations | Primary care/ Access to primary care/ 10 primary care centres | Mixed methods<br>And<br>Qualitative service evaluation | To use a QI initiative to increase the capacity of health centre to provide culturally affirming care for LGBT patients | **Generally positive results.** Improvements in documentation practices and the provision of culturally affirming care was observed. |
| Gallaher et al., 2020<br>(65) | UK | Homeless populations | Secondary care/ Emergency care/ 1 ED | Clinical note audit | To improve the quality, safety and equity of health care for homeless patients attending the ED | **Mixed results.** Compliance with homelessness care pathway processes low, but staff confidence increased. |
| Gonzalez et al., 2022<br>(66) | USA | Race | Primary care/ Covid-19 vaccination/ 1519 participants | Clinical record audit | To develop and implement an equity focused community outreach intervention to facilitate CV-19 vaccine appointments | **Mixed results.** Intervention increased vaccine rates for those who had scheduled an appointment over the phone. However, many patients who were reached declined a vaccine. |
| Green et al., 2018 | UK | Severe Mental Illness (SMI) | Secondary care/ SMI/ 318 participants | Analysis of clinical records | To improve the physical health of patients with serious mental illness | **Mixed results.** Improvements in care measures seen, but inconsistencies in care provision remained. |

| | | | | | | |
|---|---|---|---|---|---|---|
| (51) | | | | | using three interlinked QI interventions | |
| Greenwood and Shiers 2015<br><br>(49) | UK | SMI | Secondary care/ Physical fitness/ 500 participants | Mixed methods | To assess the effectiveness of the monitoring of cardiometabolic risk in patients with severe mental illness | **Generally positive results.** Rates of cardiometabolic screening rose to between 60% and 80%. Qualitative measures indicated increased staff and service user satisfaction. |
| Harrold et al., 2017<br><br>(67) | USA | Veteran health | Secondary care/ Mental illness/ 49 participants | Pre and post-test observational data | To improve the health and wellness of veterans living with severe mental illness | **Generally positive results.** Movement increased, and blood pressures became more controlled in the target group. |
| Hassaballa et al., 2015<br><br>(36) | USA | Race, gender | Community health setting/ Diabetes/ 148 participants | Mixed methods | To examine the implementation of a diabetes care program on African American women living with T2 diabetes | **Generally positive results.** mprovements in clinical indicators were seen. |

| | | | | | | |
|---|---|---|---|---|---|---|
| Martinez et al., 2021<br><br>(68) | USA | Language | Secondary care/ Emergency care/ 4573 participants | Descriptive statistics EHR | To increase interpreter use and documentation for Spanish-speaking patients attending the ER. | **Mixed results.** Use of interpreters increased, but return to the ED within 48 hours did not decrease as expected. |
| Olsson et al., 2014<br><br>(48) | Sweden | Ethnicity | Community health setting/ Cervical cancer screening/ N/A | Mixed methods | To use local doulas to raise awareness of cervical cancer screening participation | **Generally positive results.** Increased screening by 40% in target area. |
| Satti et al., 2021<br><br>(69) | USA | Age | Secondary care/ Obesity/ 885 clinical records | Descriptive statistical analysis of EHR, p-chart analyses | To improve provider adherence to care guidelines for children with obesity | **Generally positive results.** Statistically significant improvement in 3/5 QI measures. |
| Watanabe et al., 2023<br><br>(50) | USA | Age, socio-economic status | Secondary care; dental care/ Dental caries/ 315 participants | Descriptive statistics of clinical data | To ensure all children living in the study area received risk-based oral health care and preventive treatments | **Generally positive results.** Decrease in dental caries risk, and overall decrease in cavities observed. |
